# Dry Needling: A Possible Detrimental Treatment After Total Hip Arthroplasty

**DOI:** 10.7759/cureus.72053

**Published:** 2024-10-21

**Authors:** Hendrik Cornette, Patrick Demey, Luk Verhelst, Koen Liekens

**Affiliations:** 1 Orthopedic Surgery, Universitair Ziekenhuis (UZ) Leuven Gasthuisberg, Leuven, BEL; 2 Orthopedics and Traumatology, AZ Sint-Lucas Gent, Gent, BEL; 3 Orthopaedics and Traumatology, AZ Sint-Lucas Gent, Gent, BEL

**Keywords:** acupuncture therapy, dry needling, physiotherapy rehabilitation, postoperative rehabilitation, prosthetic joint infection (pji), soft tissue injuries, total hip arthroplasty (tha)

## Abstract

This case study describes a severe and rare complication following the placement of a total hip prosthesis in a 59-year-old woman. During rehabilitation, a physiotherapist incorporated dry needling alongside conventional exercises. A large ulcer developed near the surgical scar, attributed to pyoderma gangrenosum. The initiation of appropriate treatment led to a successful recovery, with no discernible impact on the current functional status. This case report aims to highlight the lack of empirical support for the use of dry needling in the post-prosthesis surgery rehabilitation process and emphasizes the associated inherent risks.

## Introduction

Total hip arthroplasty (THA) is a well-established and frequently performed procedure for alleviating symptomatic end-stage osteoarthritis of the hip [1]. Up to 97% of patients experience an improvement in their health status following the intervention, demonstrating a high satisfaction rate [2]. Post THA, a structured rehabilitation period is imperative to optimize outcomes and functional recovery. Currently, accelerated perioperative rehabilitation protocols are increasingly employed, yielding positive effects both socio-economically and in terms of patient satisfaction [3]. Standardized rehabilitation protocols typically include early mobilization initiated during the hospital stay [4], followed by a tailored program that may vary among physicians or clinics. These programs commonly incorporate treadmill exercises, resistance training targeting quadriceps muscles, interval-arm exercises, and, in later stages, weight-bearing exercises with hip-abductor eccentric strengthening [5]. Supervision of these exercises is commonly entrusted to physiotherapists.

Dry needling, a technique that uses thin needles to elicit local reactions in various tissues within a presumed neural network, is a popular technique used by physiotherapists [6]. Although widely used, this technique is not without risk. Numerous complications have been documented, ranging from skin infections to septic arthritis and, in severe cases, neural and organ damage [7]. In the case we present, the patient developed pyoderma gangrenosum, an uncommon inflammatory dermatological condition. It is characterized by the emergence of painful, rapidly expanding cutaneous ulcers with irregular edges. This condition is believed to involve a profound imbalance in components of innate and adaptive immunity [8].

## Case presentation

A 59-year-old woman presented with complaints related to her left hip. Imaging revealed degenerative hip arthritis, and the treatment involved replacing the joint with a total hip prosthesis. The patient underwent surgery and was included in the hospital-specific “rapid recovery” protocol. The hospital stay and procedure were without complications, and the patient was discharged uneventfully after two days. Mobilization and full weight bearing started a few hours postoperatively. At the first postoperative follow-up at four weeks, there were no notable issues. Both clinical and radiological assessments were satisfactory.

Six weeks postoperatively, the patient expressed concerns about a bruise near the scar. It was identified as a spider-like cluster of superficial varicose veins with local ecchymosis. An ultrasound examination revealed a limited seroma, considered a normal postoperative finding. No clinical or biochemical evidence supported the development of a periprosthetic joint infection (PJI). The wound developed after a session of dry needling during rehabilitation five weeks postoperatively.

**Figure 1:**
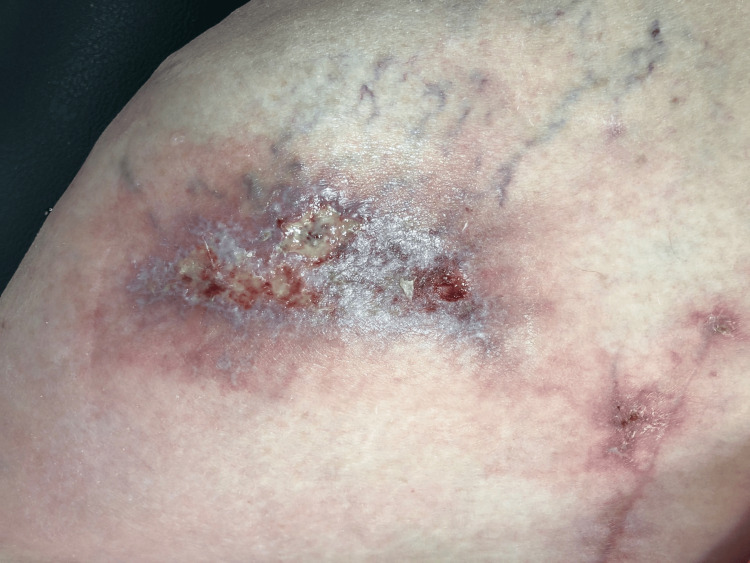
Dermatitis with flebitis and a forming ulcer The surgical scar is located in the lower right quadrant, clearly separated from the lesion.

**Figure 2:**
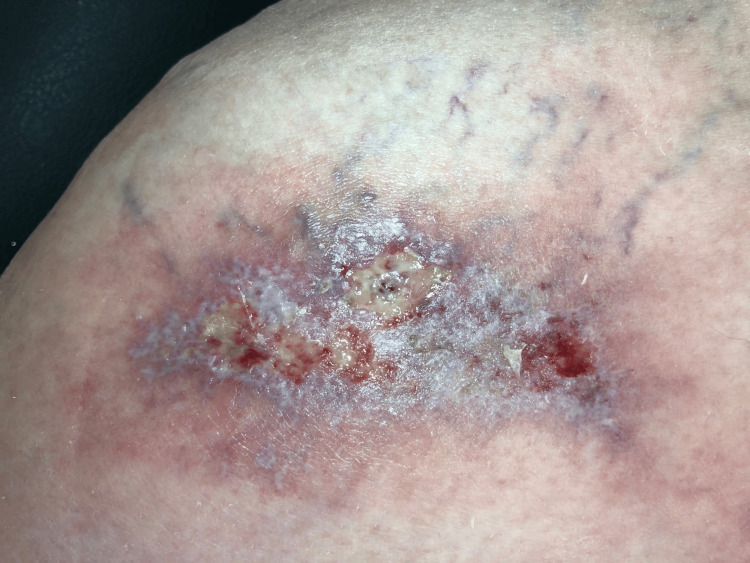
Dermatitis with flebitis and a forming ulcer

One month later, at the 10-week postoperative mark, there was further deterioration of the superficial wound. Extensive dermatitis with superficial phlebitis and a highly sensitive ulcer were observed. Due to the patient's concerns, an aspiration of the hip joint was performed, yielding negative results. Dermatology colleagues were consulted regarding the wound issue (Figures 1, 2). Initial differential diagnoses included livedoid vasculopathy, polyarteritis nodosa (PAN)-type vasculitis, and postoperative/infectious causes.

Because of clear deterioration and pronounced pain at the wound site, the patient was admitted to the dermatology department for three days. Treatment included a tapering schedule of methylprednisolone and levofloxacin. Local wound care with an alginate and antimicrobial topical cream and sterile vaseline gauze was initiated. Given the possible traumatic origin of the wound (dry needling), pyoderma gangrenosum was considered after two weeks as there was no positive evolution. Treatment was switched to cyclosporine and a topical fusidic acid and betamethasone combination. One week later, there was a remarkable improvement in the wound, confirming the diagnosis of pyoderma gangrenosum due to the significant response to cyclosporine. Currently, seven months after the total hip prosthesis, the patient is finally satisfied with both the hip and the wound.

## Discussion

Pyoderma gangrenosum is a rare skin disease (three to 10 cases per million patients) characterized by painful, rapidly evolving cutaneous ulcers. The disease is caused by complex involvement and dysregulation of innate and adaptive immunity in patients who often have an underlying predisposition, such as inflammatory bowel diseases (IBD), rheumatological disorders, hematological malignancies, or autoinflammatory syndromes. The differential diagnosis encompasses venous and arterial ulcers, medium and large vessel vasculitis, occlusive vasculopathy, ecthyma and ecthyma gangrenosum, ulcerative infections, ulcerating skin tumors and lymphomas, as well as drug-induced ulcers, including iododerma and bromoderma [8]. Treatment is typically based on topical and systemic corticosteroids, cyclosporine, and pain management [9]. To our knowledge, only one other case report of pyoderma gangrenosum complications has been published [10]. In that patient, due to dysregulation of the immune system, the surrounding tissue at the needle insertion site became necrotic, and deep ulcers formed.

After THA, a rehabilitation period is necessary for optimal outcomes and functional recovery. Several protocols are used for this purpose [3]. However, it is debatable whether these protocols always need supervision or if, with adequate patient education, an outpatient program could be implemented. Austin et al. [11] performed a single-center case series with 120 patients after primary THA. One half followed formal therapy, while the other half performed functional home exercises. There was no significant difference in any functional outcome scores, suggesting that outpatient rehabilitation is safe and efficient and that formal therapy may not always be necessary. However, not all patients benefit equally from one type of rehabilitation. It was observed that a minor group of patients initially in the outpatient group did not make progress and were transferred to the formal therapy group; typically older patients with worse preoperative functional scores. Conversely, another minor group chose to be in the outpatient group due to socioeconomic reasons. It is thus still important to create a patient-tailored program depending on individual needs.

Dry needling involves inserting thin, monofilament needles into neural, muscular, and connective tissues. In muscular tissue, needles are inserted into myofascial trigger points (MTrPs) to elicit a local reaction [6]. Other studies have shown techniques targeting structures such as ligaments, scar tissue, tendons, bone, teno-osseous insertion sites, and perineural tissue [12]. There is a theory that by inserting a needle into an MTrP, high-pressure stimulation or mechanical irritation can elicit a local twitch response (LTR), which causes a neural impulse. These nociceptors send neural impulses to the spinal cord, inducing central sensitization of the dorsal horn cells, to which MTrPs in the referred zone project, providing pain relief [13]. An MTrP is localized by palpation of a local, irritable nodule in a taut band within a muscle that causes referred pain. The diagnostic procedure for MTrPs is not standardized and has a high degree of inter-observer variability [14].

Several studies have demonstrated pain relief in various conditions, such as knee osteoarthritis, hip osteoarthritis, carpal tunnel syndrome, migraine, tension-type headache, and chronic muscular pain [12]. However, there is limited evidence of superior outcomes compared to placebo controls. Most studies have poor quality and limited sample sizes. A meta-analysis by Tough et al. concluded that there is no statistical difference between dry needling and standardized care [15].

A single case series on dry needling after THA has been published [16]. Baumann et al. described a series of two patients who were successfully treated, resulting in complete pain alleviation and an adequate range of motion. One patient suffered from inexplicable lateral pain, while the other had a fall one month prior to presentation. A combination of dry needling and physical therapy was initiated, alleviating symptoms after four months for the first patient and mild improvement for the second. Limitations of this series include the small sample size, the absence of a clear diagnosis for the pain, the lack of imaging, and the use of other therapies such as pain medication. It is not possible to conclude that dry needling has a place in THA rehabilitation based on this article [16].

Another review was written on the effect of dry needling on knee osteoarthritis [17]. Ughreja et al. performed a systematic review including nine studies. They stated that dry needling has a significant effect on short-term pain relief; however, there is no evidence of long-term benefits due to the heterogeneity of the studies and lack of quality. The results show that short-term pain relief with dry needling alone is not superior to the combination of functional exercise with dry needling or exercise with sham dry needling [17]. Further research is suggested to determine whether dry needling has a place in the treatment of knee osteoarthritis.

The effect of dry needling on hip osteoarthritis has been described in a case series by Ceballos-Laitas et al. [18]. A randomized controlled trial (RCT) was performed with two patient groups: one was treated with dry needling of the hip flexors, and the other began a home stretching exercise protocol. The philosophy is that hip extension is the most restricted movement in hip osteoarthritis, so by specifically treating this, the range of motion, stiffness, and pain will improve. The results showed improvements in both groups in extension, range of motion, stiffness, and pain. Only in extension was there a greater improvement for the dry needling group. Limitations of this study include small sample size, lack of long-term results, the two treatments being discussed in isolation, and not accounting for physical activity level and occupation [18].

Dry needling is not without complications, as demonstrated by a systematic review of case reports between 2000 and 2011 by Xu et al. [7]. Most complications are related to infections caused by needling with unsterile equipment and unhygienic procedures. Infections with *Mycobacterium *and *Staphylococcus *are most frequently described. Necrotizing fasciitis, septic arthritis, and erysipelas are documented. Other complications related to organs (such as pneumothorax and aneurysms) and nerve damage due to incorrect needle placement have also been reported [7].

## Conclusions

This case highlights the significant risks associated with incorporating dry needling into the postoperative regimen following THA. Although THA is a well-established and effective procedure for managing end-stage hip osteoarthritis, this patient encountered a severe and unexpected complication: pyoderma gangrenosum. Initially, the patient's postoperative recovery appeared routine, but the emergence of this rare dermatological condition required complex diagnostic and therapeutic intervention. Fortunately, with appropriate treatment, the patient showed substantial improvement, emphasizing the need for careful consideration of potential complications.

Despite THA's generally low complication rates, the inclusion of dry needling in post-THA rehabilitation protocols warrants critical evaluation. Some studies have suggested potential benefits, such as pain reduction and improved range of motion; however, the current evidence is limited and often inconclusive. Dry needling has been associated with a range of complications, including infections and neural damage, underscoring the necessity for cautious application of this technique. The evolving field of post-THA rehabilitation is exploring alternative modalities, including personalized outpatient programs, which may provide comparable efficacy to traditional inpatient therapy for many patients. Thus, it is crucial to balance innovative rehabilitation approaches with thorough preoperative evaluation, vigilant postoperative monitoring, and evidence-based practices to optimize recovery outcomes and minimize risks associated with interventions like dry needling. Continuous research and clinical vigilance are essential to ensure the safety and efficacy of rehabilitation strategies in the THA recovery process.
